# Changing patterns of tobacco consumption in Mozambique: evidence from a migrant study

**DOI:** 10.1186/1471-2458-11-322

**Published:** 2011-05-16

**Authors:** Nuno Lunet, Carla Araújo, Carla Silva-Matos, Albertino Damasceno, Lídia Gouveia, Ana Azevedo

**Affiliations:** 1 Department of Hygiene and Epidemiology, University of Porto Medical School, Porto, Portugal; 2 Institute of Public Health - University of Porto (ISPUP), Porto, Portugal; 3 Department of Cardiology, Centro Hospitalar de Entre o Douro e Vouga, EPE, Hospital de São Sebastião, Santa Maria da Feira, Portugal; 4 Department of Non-Communicable Diseases, Mozambique Ministry of Health, Maputo, Mozambique; 5 Faculty of Medicine, Eduardo Mondlane University, Maputo, Mozambique; 6 Department of Mental Health, Mozambique Ministry of Health, Maputo, Mozambique

**Keywords:** Tobacco, Survey, Migrants, Mozambique

## Abstract

**Background:**

Maputo, the Mozambique capital, contrasts with the rest of the country with regard to its sociodemographic characteristics and patterns of tobacco exposure. We conducted a migrant study to compare the prevalence of manufactured-cigarette smoking and traditional forms of tobacco use among dwellers in the capital who were also born in Maputo City (MC/MC) with those born in southern (SP/MC) and northern (NP/MC) provinces, and additionally with inhabitants in the latter regions.

**Methods:**

In 2003, a representative sample of 12,902 Mozambicans aged 25-64 years was evaluated. We computed age- and education-adjusted prevalence ratios (PR) with 95%-confidence intervals (95%CI) using Poisson regression.

**Results:**

The prevalence of any type of tobacco consumption among Maputo City inhabitants born in other provinces contrasted with the pattern observed in locally born inhabitants (SP/MC vs. MC/MC: men, PR, 0.61; 95%CI, 0.44-0.85; women, PR, 0.38, 95%CI, 0.18-0.79; NP/MC vs. MC/MC: men, PR, 0.66; 95%CI, 0.34-1.29; women, PR, 4.56, 95%CI, 1.78-11.69); the prevalence among city inhabitants born in other provinces resembled the pattern seen in inhabitants of their provinces of origin. Traditional forms of tobacco consumption among men were rare in Maputo City, which is in stark contrast to the situation in other provinces.

**Conclusions:**

Cultural background, affordability, and availability of different types of tobacco in urban Mozambique need to be considered when developing strategies to control the tobacco epidemic.

## Background

The patterns of tobacco consumption are shaped by demographic, economic, and cultural determinants [1], by pressure from the tobacco industry, and by the enforcement of measures to control tobacco exposure [2-4], all of which operate to a different extent in specific settings and phases of the tobacco epidemic. Transnational tobacco manufacturing and tobacco leaf companies resist tobacco control by stressing the economic importance of tobacco to the developing countries that grow it [4,5], and effective anti-tobacco policies are still lacking in many developing countries. Specifically in Mozambique, a country that has signed but not yet ratified the Framework Convention on Tobacco Control [6], tobacco production has recently increased: from 1500 tons, involving about 6000 producers, in 1997, to 60,000 tons, involving about 150,000 producers, in 2006, mostly in the north of the country [7].

In contrast to the situation in most high-income societies, the consumption of hand-rolled cigarettes and smokeless tobacco is common in the African setting [8]; however, urbanization is driving a shift from the consumption of traditional forms of tobacco to smoking manufactured cigarettes [9]. This is to be expected in Mozambique, where we recently showed that tobacco consumption is more frequent than would normally be the case for an African country at an early stage of the tobacco epidemic, especially with hand-rolled cigarettes and smokeless tobacco. Women consume predominantly smokeless tobacco, especially in the north of Mozambique; southern and urban men smoke mostly manufactured cigarettes, while those from rural areas in the north more frequently opt for hand-rolled cigarettes [10].

Therefore, we hypothesized that among the dwellers in each Mozambican region, there might be differences in the patterns of consumption according to the place of birth, and we tested this hypothesis with a migrant-study design. We aimed to compare the prevalence of manufactured-cigarette smoking and traditional forms of tobacco use among dwellers in the capital, Maputo City, who were born in the capital with Maputo inhabitants born in other southern or northern regions of Mozambique, and additionally with inhabitants of the same southern and northern regions.

## Methods

### Study design

Between September and December 2003, 12,902 subjects aged 25 to 64 years were evaluated in a community-based cross-sectional study, using the sampling frame of the 1997 census, to be representative at the national and provincial levels as well as by place of residence (urban or rural). We selected 604 geographical clusters (68 in each of the two largest provinces and 52 in each of the remaining nine provinces) out of the 44,931 clusters that cover the whole Mozambican territory. In each geographical cluster, all the households were listed (mean number of households per cluster, 103; range, 80-150) and 24 were randomly selected and visited. Homeless individuals and people living in collective residential institutions (e.g., hotels, hospitals, military facilities) were ineligible. All the eligible subjects in the same household were invited to participate in the study.

The definition of the sampling weights used in the data analysis was based on the best estimates of the population in each primary sampling unit at the time the study was designed. The sampling weights were then corrected for participation at the household level in each geographical cluster and taking into account the variation in the size and age structure of the population (according to the official projections of the National Institute of Statistics of Mozambique) for the population living in each province when the study was conducted.

### Data collection

Trained interviewers conducted face-to-face interviews in each household. Given the ethnic and linguistic diversity in Mozambique, all interviewers were able to correctly speak the predominant languages in the regions where they collected data. Subjects were evaluated following standardized procedures and using a structured questionnaire for assessing sociodemographic and behavioral factors [11].

The use of any tobacco product, including manufactured cigarettes, hand-rolled cigarettes, and smokeless tobacco, was assessed in all subjects. Current tobacco consumers were asked about the type of tobacco most often used and classified as consumers of manufactured cigarettes or of traditional forms of tobacco use (hand-rolled cigarettes or smokeless tobacco). Only 20 participants reported cigar or pipe smoking as their predominant type of tobacco consumption, and these categories were not considered in the analysis of type of tobacco use.

The place of residence was classified as urban (in any of the 23 cities and 68 towns) or rural (outside cities or towns) [12]. Education was registered as the highest education level attained, and participants were grouped in four categories (<1 year, 1-5 years, 6-7 years, ≥8 years) according to the years of schooling.

**Figure 1:**
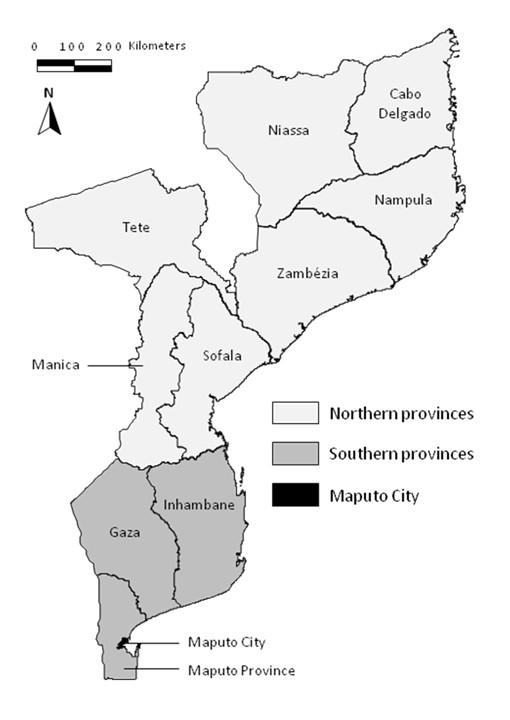
**Distribution of Mozambican provinces and grouping for data analysis**.

The province of Maputo City is the only one of the nation's 11 provinces that hosts a high proportion of internal migrants (Table 1); it also presents the strongest contrasts with the rest of the country in terms of sociodemographic characteristics (Table 1) and smoking habits of the population [10]. Therefore, for our analysis, we grouped participants according to their place of residence, as shown in Figure 1: Maputo City (1,162,000 inhabitants in an area of 633 km^2^); other southern provinces (3,561,000 inhabitants in 170,283 km^2^); northern provinces (13,789,000 inhabitants in 629,276 km^2^) [13]. The dwellers in the capital were further classified according to their place of birth: Maputo City; other southern provinces; northern provinces.

**Table 1:** Sociodemographic characteristics of the participants.

	Place of residence in 2003					
	
	Maputo City		Southern provinces^a^		Northern provinces^b^	
			
	N	Weighted %	n	Weighted %^c^	n	Weighted %^c^
**Women**	**724**		**2368**		**4756**	

Place of birth						
Maputo City	282	38.3	70	3.0	7	0.0
Southern provinces^a^	408	57.0	2256	95.2	57	0.9
Northern provinces^b^	23	3.7	36	15.5	4581	96.9
Out of the country	11	1.0	6	0.2	105	2.2
Place of residence in 1998^d^						
Maputo City	667	93.1	54	2.2	2	0.0
Southern provinces^a^	43	6.1	2284	97.1	3	0.1
Northern provinces^b^	5	0.8	13	0.7	4722	99.8
Other country	^e^	^e^	^e^	^e^	5	0.1
Place of residence in 2003						
Urban	724	100.0	873	33.0	1274	21.9
Rural	0	0.0	1495	67.0	3482	78.1
Age (years)						
25-34	291	40.3	864	37.0	1996	42.2
35-44	215	29.5	646	27.6	1255	25.4
45-54	150	20.2	507	20.8	950	20.6
55-64	68	9.9	351	14.6	555	11.8
Education (years)						
<1	110	14.4	1011	43.7	2819	61.6
1-5	402	54.6	1085	45.1	1637	33.7
6-7	124	17.7	191	8.0	186	3.0
≥8	88	13.2	80	3.2	101	1.7

**Men**	**393**		**1053**		**3597**	

Place of birth						
Maputo City	143	37.5	34	3.2	1	0.0
Southern provinces^a^	205	52.1	955	91.8	30	0.6
Northern provinces^b^	40	9.3	58	4.7	3500	98.0
Out of the country	5	1.1	6	0.2	61	1.4
Place of residence in 1998^d^						
Maputo City	345	92.8	35	3.0	4	0.0
Southern provinces^a^	15	4.1	967	95.0	5	0.2
Northern provinces^b^	11	3.1	12	1.1	3539	99.6
Other country	^e^	^e^	^e^	^e^	5	0.1
Place of residence in 2003						
Urban	393	100.0	401	34.1	902	20.1
Rural	0	0.0	652	65.9	2695	79.8
Age (years)						
25-34	153	39.3	324	28.9	1323	37.5
35-44	99	25.5	278	27.2	1020	27.7
45-54	83	19.2	249	24.1	756	20.7
55-64	58	16.0	202	19.8	498	14.2
Education (years)						
<1	12	2.3	217	20.4	903	28.6
1-5	211	52.6	569	55.9	2022	55.2
6-7	90	23.5	153	14.6	413	10.5
≥8	80	21.5	114	9.1	254	5.7

^a ^Maputo Province, Gaza and Inhambane; ^b ^Manica, Sofala, Tete, Zambézia, Nampula, Niassa and Cabo Delgado; ^c ^Within each variable the sum of the proportions may not be 100% due to rounding; ^d ^The sum of the number of participants in each category is lower than the total in each group due to missing data; ^e ^No observations.

### Statistical analysis

We computed age- and education-adjusted prevalences and prevalence ratios (PR) with 95%-confidence intervals (95%CI) using logistic and Poisson regression models, respectively. The following groups were defined for data analysis, according to the place of birth/place of residence: Maputo City/Maputo City (MC/MC); southern provinces/Maputo City (SP/MC); southern provinces/southern provinces (SP/SP); northern provinces/Maputo City (NP/MC); northern provinces/northern provinces (NP/NP). All the analyses were conducted considering the sampling weights and adjusting for strata and clustering at the primary-sampling-unit level using Stata^® ^(StataCorp, College Station, Texas, USA), version 9.2, and included 12,891 subjects (complete data were not available for only 11 subjects). The study protocol was approved by the National Mozambican Ethics Review Committee, and written informed consent was obtained from all participants.

## Results

### Characteristics of the study sample

Among the dwellers in Maputo City, nearly three-quarters were born in other provinces, predominantly in the south; however, more than 90% had already been living in Maputo for five or more years before the survey was undertaken. Fewer than 10% of the inhabitants of southern provinces and fewer than 5% of those living in the north were born in other regions. Maputo City is an exclusively urban area, and the proportion of subjects living in rural settings was higher in the northern provinces (approximately three-quarters) than in southern ones (approximately three-fifths) (Table 1).

Most subjects were under 45 years of age (two-thirds); there were no meaningful differences by region, except for a higher proportion of older subjects among those living outside Maputo City. Approximately half the women and one-quarter of men had no formal education. The proportion of subjects with the lowest education levels gradually increased from Maputo City (15.2% among women, 3.0% among men) to the north (59.4% among women, 25.1% among men) (Table 1).

### Tobacco consumption according to internal migration to Maputo City

**Figure 2:**
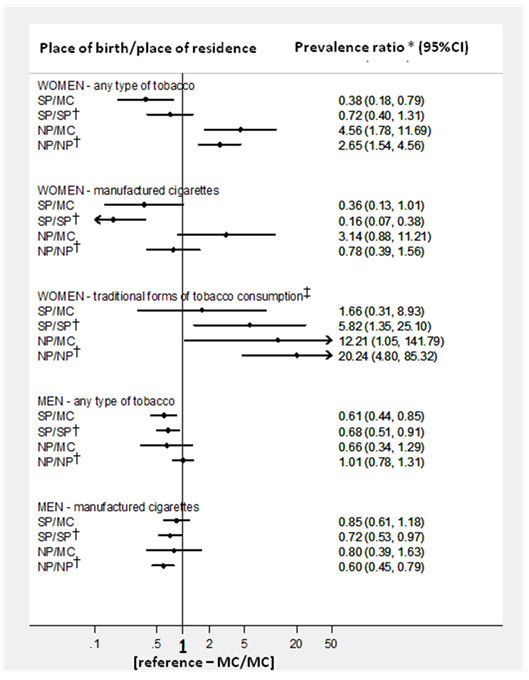
**Prevalence ratios for tobacco consumption for different combinations of place of birth/place of residence (in 2003) compared with participants born and living in Maputo City**. 95%CI - 95% confidence interval; MC - Maputo City; NP - Northern provinces (Manica, Sofala, Tete, Zambézia, Nampula, Niassa and Cabo Delgado); SP - Southern provinces (Maputo Province, Gaza and Inhambane). * Age- and education-adjusted prevalence ratios for tobacco consumption, having the subjects born and living in Maputo City as the reference; † These groups include a small proportion of subjects born elsewhere; ‡ Traditional forms of tobacco consumption include hand-rolled cigarettes and smokeless tobacco.

The prevalence estimates for tobacco consumption in each subgroup are presented in Table 2. Figure 2 depicts the corresponding PR using MC/MC as the reference category.

**Table 2:** Prevalence of different forms of tobacco consumption among participants living in Maputo City according to their place of birth and among participants living in other Mozambican provinces.

		Tobacco consumption								
		
	All participants		Any type of tobacco			Manufactured cigarettes^a^			Traditional forms of tobacco consumption^a^	
				
	N	n	Crude weighted prevalence % (95%CI)	**Adjusted**^b ^**weighted prevalence % (95%CI)**	N	Crude weighted prevalence % (95%CI)	**Adjusted**^b ^**weighted prevalence % (95%CI)**	n	Crude weighted prevalence % (95%CI)	**Adjusted**^b ^**weighted prevalence % (95%CI)**
**Women**										

Place of birth/place of residence										
Maputo City/Maputo City (MC/MC)	282	14	4.4 (1.8-6.9)	6.4 (3.7-11.0)	12	4.0 (1.5-6.5)	4.4 (2.3-8.0)	2	0.4 (0.0-1.0)	0.6 (0.1-2.4)
Southern provinces^c^/Maputo City (SP/MC)	408	12	3.1 (0.8-5.4)	2.0 (0.9-4.3)	4	1.6 (0.0-3.6)	1.5 (0.4-4.7)	8	1.4 (0.1-2.8)	0.9 (0.3-2.2)
Southern provinces^c,d^/Southern provinces^c ^(SP/SP)	2366	138	6.3 (4.4-8.2)	3.9 (2.8-5.3)	16	0.7 (0.3-1.1)	0.6 (0.3-1.2)	121	5.6 (3.7-7.4)	3.2 (2.2-4.5)
Northern provinces^e,d^/Maputo City (NP/MC)	23	4	19.6 (16.3-37.6)	32.0 (13.1-59.4)	3	16.2 (0.0-33.7)	16.8 (5.2-42.6)	1	3.4 (0.0-10.1)	6.4 (0.9-33.4)
Northern provinces^e,d^/Northern provinces^e ^(NP/NP)	4749	1003	23.1 (20.9-25.2)	18.1 (15.9-20.5)	126	3.3 (2.2-4.3)	3.2 (2.4-4.3)	868	19.6 (17.6-21.6)	13.8 (11.9-16.1)

**Men**										

Place of birth/place of residence										
Maputo City/Maputo City (MC/MC)	143	50	35.2 (26.2-44.2)	42.2 (32.7-52.4)	50	35.2 (26.2-44.2)	26.0 (18.7-35.0)	0	f	f
Southern provinces^c^/Maputo City (SP/MC)	205	51	24.4 (19.3-29.4)	26.6 (21.4-32.4)	49	23.0 (17.8-28.2)	22.2 (17.1-28.3)	1	0.8 (0.0-2.5)	0.9 (0.1-5.9)
Southern provinces^c,d^/Southern provinces^c ^(SP/SP)	1053	295	28.1 (24.5-31.8)	29.1 (25.5-33.0)	205	19.1 (16.3-21.9)	18.4 (15.7-21.5)	87	8.4 (5.8-11.0)	7.7 (5.7-10.4)
Northern provinces^e^/Maputo City (NP/MC)	40	12	24.2 (9.5-38.9)	28.5 (15.2-47.0)	12	24.2 (9.5-38.9)	20.3 (9.6-38.0)	0	f	f
Northern provinces^e,d^/Northern provinces^e ^(NP/NP)	3595	1548	42.7 (40.5-45.0)	43.4 (41.2-45.7)	567	16.2 (14.2-18.1)	15.2 (13.3-17.2)	973	26.3 (23.7-28.9)	25.6 (23.0-28.4)

95%CI - 95% confidence interval.

^a ^Form of tobacco consumption reported as the most frequent by current smokers; ^b ^Age- and education-adjusted; ^c ^Maputo Province, Gaza and Inhambane; ^d ^These groups include a small proportion of subjects born elsewhere; ^e ^Manica, Sofala, Tete, Zambézia, Nampula, Niassa and Cabo Delgado; ^f ^No observations.

#### Women

The consumption of any type of tobacco was lower in SP/MC than in MC/MC (adjusted prevalences, 2.0% vs. 6.4%; adjusted PR, 0.38), but this does not apply to the traditional forms of tobacco use (adjusted prevalences, 0.9% vs. 0.6%; adjusted PR, 1.66).

The frequency of manufactured-cigarette smoking was much lower in SP/SP than in MC/MC (adjusted prevalences, 0.6% vs. 4.4%; adjusted PR, 0.16), and an association of similar magnitude, but in the opposite direction, was observed for the traditional forms of tobacco use (adjusted prevalences, 3.2% vs. 0.6%; adjusted PR, 5.82).

When comparing SP/MC with SP/SP, no statistically significant differences were observed for the consumption of any type of tobacco (adjusted PR, 0.53; 95%CI, 0.25-1.13) or manufactured-cigarette smoking; however, the use of traditional forms of tobacco was threefold lower in SP/MC (adjusted PR, 0.33; 95%CI, 0.12-0.88).

The prevalence of tobacco consumption was higher in NP/MC than in MC/MC (adjusted prevalences, 32.0% vs. 6.4%; adjusted PR, 4.56), and the same pattern was observed for traditional forms of tobacco use (adjusted prevalences, 6.4% vs. 0.6%; adjusted PR, 12.21). The use of traditional forms of tobacco was also more frequent in NP/NP than in MC/MC (adjusted prevalences, 13.8% vs. 0.6%; adjusted PR, 20.24).

Manufactured-cigarette smoking was more frequent in NP/MC than in NP/NP (adjusted PR, 6.14; 95%CI, 1.84-20.50), and the traditional forms were used less often in NP/MC than in NP/NP (adjusted PR, 0.87; 95%CI, 0.83-0.92).

#### Men

The consumption of any type of tobacco in SP/MC was lower than in MC/MC (adjusted prevalences, 26.6% vs. 42.2%; adjusted PR, 0.61), but it was similar to that observed in residents in the remaining southern regions (SP/MC vs. SP/SP, adjusted PR, 0.93; 95%CI, 0.72-1.20). No significant differences were observed for manufactured-cigarette smoking between SP/MC and MC/MC (adjusted prevalences, 22.2% vs. 26.0%; adjusted PR, 0.85) or SP/SP (adjusted PR, 1.20; 95%CI, 0.90-1.58). However, no traditional forms of tobacco use were observed among MC/MC, while SP/MC were less likely than SP/SP to report hand-rolled or smokeless tobacco as the most frequently type of tobacco consumption (adjusted PR, 0.14; 95%CI, 0.02-0.97).

The prevalence of tobacco consumption among NP/MC was not significantly different from that observed in MC/MC (adjusted prevalences, 28.5% vs. 42.2%; adjusted PR, 0.66) or NP/NP (adjusted PR, 0.66; 95%CI, 0.36-1.20), and the same was observed for manufactured-cigarette smoking (NP/MC vs. MC/MC, adjusted PR, 0.80; 95%CI, 0.39-1.63; NP/MC vs. NP/NP, adjusted PR, 1.30; 95%CI, 0.66-2.53). However, the prevalence of traditional forms of tobacco use among Maputo City dwellers born in the north (NP/MC) and among those born in Maputo City (MC/MC) was 0%, which is in stark contrast with the high consumption in the north (NP/NP) (adjusted prevalence, 25.6%).

## Discussion

Among Maputo City residents, there were important differences in the patterns of tobacco consumption according to the place of birth. The prevalence of manufactured-cigarette smoking in subjects born in other regions tended to differ from the pattern observed in those who were locally born, and it resembled more closely what was observed in inhabitants of their provinces of origin. More dramatic changes in the patterns of consumption with migration were observed with regard to traditional forms of tobacco use among men: there were virtually no instances of this being cited as the main type of tobacco consumed, which is in stark contrast with what was observed in other provinces.

Migrant studies have supported the role of environmental exposure as a determinant of different diseases [14-16] by showing changes in their frequency among migrant populations towards that observed in the host setting. The evidence provided by studies focusing on disease patterns according to migration status is based on the assumption that changes in the frequency of these conditions within one or two generations are predominantly explained by variations in the individual exposure to environmental factors [17]; this is supported by migrant studies that specifically address changes in behavior, such as diet or tobacco consumption [18-22].

Place of birth and place of residence are predictors of both health exposures and outcomes [21]. Characterization of the patterns of change in exposure to risk factors for chronic diseases according to internal migration, namely from rural to urban areas (in sub-Saharan Africa, it is estimated that 35% of the population lived in urban areas in 2005 and that by 2050 this proportion will have risen to 61% [23]), may support locally grounded public health interventions. Luo tribe members moving from rural land in western Kenya to urban Nairobi showed an increase in mean systolic blood pressure and a greater prevalence of hypertension after migration than tribe members who did not migrate [24]. Similarly, migrants from southwestern rural China to urban Xichang City showed an increase in serum total cholesterol [25]. In Tanzania, 12 months after migration from the Morogoro rural region to urban Dar es Salaam, migrants decreased physical activity, increased weight, and increased their intake of red meat, but also of fresh fruit and vegetables, while showing mixed changes in lipid profile and a decrease in blood pressure [26].

Studies comparing the prevalence of smoking between migrants and host populations showed that the former tend to acquire habits similar to those observed among the latter, despite the differences according to ethnic group, gender, socioeconomic group, and length of residence [27-29]. A converging prevalence of smoking was observed among Turkish men migrating to the Netherlands, while second-generation Turkish women migrants smoked significantly more than ethnic Dutch women. This trend was not observed with smoking among Moroccan migrants to the Netherlands [22]. In another study that investigated whether differences in smoking behavior between Turkish migrants and the German host population diminished with time since migration, the prevalence of tobacco consumption partly approached that of the host population, or was even higher, but only in second-generation migrants [30].

These studies report on tobacco use among various populations of immigrants, and a comparison with the results from our study should be undertaken cautiously since internal migration has specific characteristics. The process of acculturation--a key determinant of smoking behavior [31]--is less intense because the internal-migration process does not always involve a radical cultural modification, though immigration usually does [20]. Overall, internal migration studies showed that the migratory lifestyle is associated with smoking initiation [31,32] for a number of reasons related with adaptation to urban life including solitude [31,33] and unstable living situations and employment opportunities [33-35], as well as higher levels of disposable income [32,33]. Among rural-to-urban migrant workers in China, the prevalence of smoking was higher subsequent to migration compared with before (28.4% vs. 20.8%; *p *< 0.01) [31]. In another study, female migrants showed an approximately 10-fold higher prevalence of cigarette smoking than the national prevalence for the same age range, while among men the cigarette-smoking prevalence was comparable or even slightly lower than the national prevalence [32]. In both studies, cigarette smoking among migrants was related to length of stay. In the former study, the post-migration daily smoking initiation prevalence was 4.5% and 16.2% (*p *< 0.01) among those with less than 5 years and 15 or more years of residence, respectively [31]. In the second study, in which migrants reported an average of 4 years of post-migration residence, a higher smoking prevalence was observed during the first 6 months after migration; this was followed by a decline to the lowest level during the second 6 months and an increase to an asymptote as the length of stay increased [32].

It must be stressed that only manufactured-cigarette smoking was considered in the above studies. In our study, more than 90% of participants had already been living in Maputo City for at least 5 years before data collection; thus, they had migrated before the more recent undergoing urbanization process in the capital, and probably for them the cultural transition was not so abrupt. In fact, migration to Maputo represents more than a change from a less urbanized to a more urbanized setting. The regional differences in tobacco production--with a substantially higher production and greater access to raw tobacco products in the north--help explain why migrants to Maputo City tended to smoke manufactured cigarettes more often and use traditional forms of tobacco consumption less often than inhabitants of their provinces of origin. This change in the main type of tobacco consumed was very pronounced among women from the north. It probably reflects the fact that matrilineal systems, with high female illiteracy, predominate in the northern provinces, while southern women tend to receive more formal education and have greater access to information [36]. This underlines the fact that women from settings where tobacco consumption is locally acceptable are potential new consumers of manufactured cigarettes [10], and it is in accordance with this evidence that tobacco companies are increasing their targeting of women in developing countries [37].

To our knowledge, the present investigation is the first to address this topic in a sub-Saharan African country at the early stages of epidemiological transition, and it adds original and methodologically sound evidence on the dynamics of tobacco exposure in these settings to previous research efforts. It is, however, limited by the small number of internal migrants to Maputo born in the northern provinces and because the exposure to each form of tobacco use was not specifically quantified (although valid estimates were obtained regarding the exposure to different types of tobacco reported as that most frequently consumed). Furthermore, although age- and education-adjusted estimates were computed in a gender-stratified analysis, these variables are unlikely to capture fully the potential confounding effect of socioeconomic and cultural differences between migrants and the remaining population. However, it seems unlikely that the observed patterns (which are characterized by consistent relations that are in accordance with the heterogeneity in sociodemographic and cultural characteristics and tobacco-consumption patterns across regions in Mozambique) would disappear if a finer adjustment for potential confounders could have been accomplished.

## Conclusions

Setting-specific factors related to cultural background, affordability, and availability of different types of tobacco are strong determinants of tobacco-consumption patterns in urban Mozambique and need to be considered when developing strategies to tackle the progression of the tobacco epidemic. Of particular concern are the trends in smoking behavior among women.

## Competing interests

The authors declare that they have no competing interests.

## Authors' contributions

NL raised the hypotheses, analyzed and interpreted the data, and drafted the first version of the manuscript. CA raised the hypotheses, analyzed and interpreted the data, and participated in elaborating the first draft of the manuscript. CSM revised the final version of the manuscript. AD analyzed the data and revised the final version of the manuscript. LG revised the final version of the manuscript. AA analyzed and interpreted the data and participated in elaborating the first draft of the manuscript. All authors read and approved the final manuscript.

## Pre-publication history

The pre-publication history for this paper can be accessed here:

http://www.biomedcentral.com/1471-2458/11/322/prepub
